# A method for identifying alternative or cryptic donor splice sites within gene and mRNA sequences. Comparisons among sequences from vertebrates, echinoderms and other groups

**DOI:** 10.1186/1471-2164-10-318

**Published:** 2009-07-16

**Authors:** Katherine M Buckley, Liliana D Florea, L Courtney Smith

**Affiliations:** 1 The Department of Biological Sciences, Washington University, Washington, DC 20052, USA; 2 The Department of Computer Science, George, Washington University, Washington, DC 20052, USA; 3 Current address : Department of Immunology, University of Toronto, Sunnybrook Health Sciences Centre, 2075 Bayview Avenue, Toronto, Ontario, M4N 3M5, Canada; 4 Current address : Center for Bioinformatics and Computational Biology, University of Maryland 3119 Biomolecular Sciences Bldg (#296), College Park, MD 20742, USA

## Abstract

**Background:**

As the amount of genome sequencing data grows, so does the problem of computational gene identification, and in particular, the splicing signals that flank exon borders. Traditional methods for identifying splicing signals have been created and optimized using sequences from model organisms, mostly vertebrate and yeast species. However, as genome sequencing extends across the animal kingdom and includes various invertebrate species, the need for mechanisms to recognize splice signals in these organisms increases as well. With that aim in mind, we generated a model for identifying donor and acceptor splice sites that was optimized using sequences from the purple sea urchin, *Strongylocentrotus purpuratus*. This model was then used to assess the possibility of alternative or cryptic splicing within the highly variable immune response gene family known as *185/333*.

**Results:**

A donor splice site model was generated from *S. purpuratus *sequences that incorporates non-adjacent dependences among positions within the 9 nt splice signal and uses position weight matrices to determine the probability that the site is used for splicing. The *Purpuratus *model was shown to predict splice signals better than a similar model created from vertebrate sequences. Although the *Purpuratus *model was able to correctly predict the true splice sites within the *185/333 *genes, no evidence for alternative or trans-gene splicing was observed.

**Conclusion:**

The data presented herein describe the first published analyses of echinoderm splice sites and suggest that the previous methods of identifying splice signals that are based largely on vertebrate sequences may be insufficient. Furthermore, alternative or trans-gene splicing does not appear to be acting as a diversification mechanism in the *185/333 *gene family.

## Background

Genome sequencing has generated large amounts of sequence data, and the computational challenge of identifying genes, promoters, repeats and other sequence patterns has proved demanding [1-3]. Average human genes are 28 kb long, with 8.8 exons of ~120 nucleotides (nt) in length that are separated by 7.8 introns [4]. Identification of the splicing signals that flank exon boundaries involves finding the correct site amid thousands of incorrect sites. Although computationally distinguishing bulk coding from non-coding sequence has met with relative success, gene prediction programs typically identify exon boundaries with less accuracy [5]. A number of gene-finding strategies have been developed, including comparison of genomic and expressed sequence tag (EST) sequences [6], hidden Markov models to identify splicing motifs [7,8], Bayesian networks [9], inter-species genome comparisons to identify genes with homologous structures [10], and various combinations of these and other strategies [11-13]. Additionally, some progress has been made in examining exon splice sites directly in hopes of improving the accuracy of predicting these sites [14]. Nearly all of these models have been generated using sequences isolated from either mammals (primarily human and mouse), *Arabidopsis*, or yeast. As the number of sequenced invertebrate genomes increases, the need for splice site identification models designed specifically for those organisms also increases (reviewed in [15]). Despite the growing number of invertebrate genomes, little data exist to address the accuracy of employing vertebrate-based models for splice site identification in other organisms. The traditional strategies may fall short, given that novel genes have been identified in invertebrates that lack the level of similarity to known sequences required for many of the gene finding programs, and many gene prediction programs perform poorly in the GC-poor sequences [5] that may characterize invertebrate genomes [16].

Sea urchins are members of the Echinoderm phylum and provide a useful and interesting phylogenetic perspective as an invertebrate at the base of the deuterostome lineage. Although the genome of the purple sea urchin, *Strongylocentrotus purpuratus *was only recently sequenced [16], the species has long served as a model for embryonic development [17]. A number of interesting attributes of this species have been uncovered that set it apart from vertebrate or insect genomes. The GC content of the sea urchin genome is 36.9%, lower than most vertebrates [16]. The *S. purpuratus *genome is estimated to contain 23,300 genes, many of which have chordate or protostome homologues, in addition to many novel genes [16]. The average sea urchin gene structure is similar to those in humans and is 7.7 kb long, with 8.3 exons that are 100–115 nt long, and 7.3 introns that are ~750 nt long [16]. Little is known about the evolutionary conservation of the splicing proteins or the splicing signals in sea urchin gene models, however, ESTs matching splicing proteins are upregulated in sea urchin immune cells, or coelomocytes, in response to immune challenge [18] and a number of gene models encoding splicing proteins have been annotated [16]. It was therefore of interest to develop a model that could identify more accurately splicing signals within *S. purpuratus *genomic sequence.

The sea urchin also has a surprisingly complex immune system [19] consisting of a rudimentary complement system [20] and a number of significantly expanded gene families encoding proteins that are homologous to known vertebrate immune proteins [21]. The *185/333 *gene family from the purple sea urchin is an intriguing example of diversification of a family of invertebrate immune response genes [18,22-26]. The *185/333 *genes are highly expressed in response to challenge with whole bacteria [27], lipopolysaccharide (LPS; [18]), double-stranded (dsRNA), and the fungal signature β-1,3-glucan [25]. Of the 689 transcripts that have been isolated from 14 animals, 437 have unique sequences [25,26]. The diversity takes the form of substantial levels of point mutations, in addition to the presence or absence of numerous short blocks of shared sequence called *elements*. The variable presence/absence of these elements defines *element patterns*, of which 35 are currently known [23,25,26]. Multiple sequence repeats within the messages and genes allow multiple alignments that are different but equally optimal, of which two have been analyzed extensively [23].

Although initial reports speculated that the variation in element pattern might be the result of extensive alternative splicing [18], the first identification of a few *185/333 *genes in an early assembly of the sea urchin genome showed that the genes are small and composed of only two short exons [23,26]. The first exon encodes the hydrophobic leader sequence, while the second exon encodes the remainder of the open reading frame [4], including the variable element pattern (see Additional file 1A). Further analysis of the *185/333 *gene family from individual sea urchins suggests that it may be composed of 80–120 exceptionally diverse alleles [22,23,26]. Of the 215 genes that have been cloned and sequenced from four different animals, 135 have distinct sequences. The genes are flanked by di- and trinucleotide repeats, and some are linked as closely as ~3 kb [23]. This close spacing of genes is believed to promote diversification through gene duplication (unpublished data) and frequent recombination [22]. Analysis of sequences isolated from individual animals, however, indicates that the genes and messages are not identical and suggests possible cytidine deaminase-like post-transcriptional editing of the *185/333 *transcripts [24]. Although the two exon gene structure initially ruled out alternative splicing [23,26], it is possible that the discord between the gene and message sequences, particularly that of non-matching element patterns in the genes vs. messages from individual sea urchins, may be the product of cryptic splice sites within the *185/333 *genes. Two possibilities exist: cryptic splice sites within the genes i) alter the element patterns of the transcripts by removing whole elements, or ii) promote trans-gene splicing to form messages that are hybrids of two closely linked genes. It was therefore of interest to investigate putative cryptic splice sites within the *183/333 *genes to determine whether alternative or trans-gene splicing could further increase the diversity of the *185/333 *transcripts.

The results presented here test a donor splice site model that was generated using known sea urchin splice sites and is called the *Purpuratus *splice site model. It incorporates non-adjacent dependencies among positions within the donor splice site [28] and uses position weight matrices to assess the probabilities of each nucleotide (nt) in each splice site position [29]. This method is more accurate for predicting splice sites from sea urchin sequences than a similar model constructed using vertebrate sequences [28]. The *Purpuratus *model also out-performed the Vertebrate model in predicting splice sites in protostome sequences. Although putative donor cryptic splice sites were identified within the *185/333 *gene sequences, no transcripts have been identified that utilize these splice sites to either delete elements or to promote trans-gene splicing.

## Results

### Model procedure: donor splice site

Although previous research identified sequences that might act as alternative or cryptic donor splice sites from vertebrate genes and mRNAs [1-3], little attention has been paid to this area for sequences from non-model organisms. Therefore, a computational model was generated with the aim of predicting splice sites, including cryptic sites, in processed mRNAs from echinoderm sequences. The model incorporated observed dependencies among non-adjacent positions within the splice site [28] and utilized the normalized position frequency matrices (PFM) as reviewed in [29] to score each site. The first step in the splicing process occurs when the U1 small nuclear ribonuclear particle (snRNP) anneals to the donor site through base-pairing between the U1 snRNA and the bases of the donor splice site in the pre-processed message. The donor splice site consensus region consists of the last three nt of the exon (positions -3 to -1, Table 1), and the first six nt of the intron beginning with the canonical GT (positions 1 to 6). This base-pairing is degenerate, with mismatches between the U1 snRNA and a suboptimal donor sequence at some positions [28]. A model to identify these suboptimal sequences was generated using the maximal dependence decomposition (MDD) method described in [28], which identified dependencies between non-adjacent positions within the donor splice sites.

**Table 1:** Dependence between nucleotide positions in sea urchin donor splice sites.

		*j*							
Consensus^1^	*i*	-3	-2	-1	+3	+4	+5	+6	Sum (*S*_*i*_)^2^
a/c	-3	-	69.52*	25.55*	9.26	18.04*	28.16*	19.43*	169.96*
A	-2	239.52*	-	61.65*	46.64*	65.36*	118.23*	88.86*	620.26*
G	-1	17.87*	153.74*	-	45.83*	90.60*	106.86*	163.67*	578.57*
A	+3	0.57	4.81	14.81	-	23.08*	92.18*	57.77*	193.22*
A	+4	26.19*	68.12*	90.26*	99.15*	-	33.43	15.2	332.35*
G	+5	34.95*	126.64*	104.82*	89.73*	94.86*	-	146.11*	597.11*
T	+6	34.68*	94.79*	162.78*	48.67*	2.77	103.4*	-	447.09*

^1^Positions 1 and 2 (the canonical GT site) were omitted because they were invariant.

^2^The sum of the χ^2 ^values (*S*_*i*_; Equation 1) is shown in the column on the left.

*Indicates significant values (P < 0.001)

**Figure 1:**
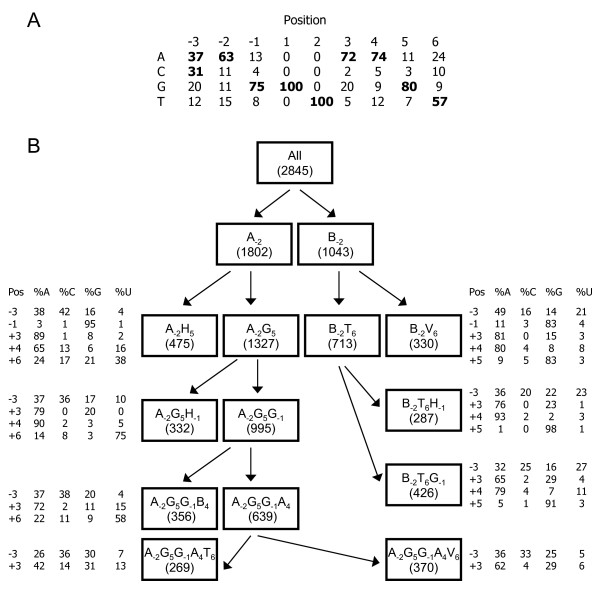
***Purpuratus *donor splice site model**. **A**. Analysis of the frequency of each base within the splice site reveals the *S. purpuratus *donor splice site consensus sequence. The nine nt window surrounding the donor splice sites from 292 annotated *S. purpuratus *gene models (2845 donor sequences) were extracted, and the frequency of each nt within the window was calculated. The values shown in bold are the consensus nucleotides. Positions 1 and 2 are invariant because only canonical splice sites were used in this analysis. **B**. The *Purpuratus *splice site model incorporated non-adjacent dependences among the bases within the splice site. The model is implemented such that a splice site score of a given candidate sequence is computed using the matrix determined by applying the set of rules shown in the flowchart. For example, the sequence A**AG**GTA**AGT **would be scored using the matrix A_-2_G_5_G_-1_A_4_T_6 _(A_-2_→A_-2_G_5_→A_-2_G_5_G_-1_→ A_-2_G_5_G_-1_A_4_→A_-2_G_5_G_-1_A_4_T_6_). Non-adjacent dependences were calculated for the 2845 *S. purpuratus *donor splice sites for each of the seven variable positions between the consensus nt and the non-consensus nucleotides in the other six positions (Table 1). The position with the maximum dependencies was used to serially subdivide the sites until either the subdivision became too small to obtain reliable data, or no more significant dependences were observed. Position frequency matrices are shown, which were calculated for each of the terminal subdivisions and ultimately used in the *Purpuratus *splice site model.

Two models were generated: one using vertebrate sequences as the source for the dependencies and PFMs, and a second that used annotated *S. purpuratus *genes. The Vertebrate model was constructed using the model and nt frequencies shown in [28]. A set of 292 annotated *S. purpuratus *gene models [16] containing at least two exons was collected (January, 2007; these sequences constituted the "*Purpuratus *model" sequence set; Table 2). In total, 2,845 donor splice sites were extracted from the sequences (Table 2) and the frequency of the nt in each position was calculated to generate a consensus sequence (Figure 1A). Splice site sequences containing N's were omitted from the analysis. For each of the seven variable positions within the donor splice site, the significance of the dependencies was calculated between the consensus nucleotide and the non-consensus nucleotides in the other six positions (see Table 1 for an example of a dependencies table). Although the donor site is traditionally defined as positions -3 to +6 relative to the GT site, the nucleotide frequencies were calculated from -20 to +20. Additional sequence may contribute to the spliceosome specificity; however, incorporating data from these positions did not affect the accuracy of the model presented here (data not shown). Significant dependencies were used to subdivide serially a set of known donor splice sites to generate the model (Figure 1B). After the data were completely partitioned (see Methods), PFMs were generated for each of the subdivisions by determining the frequency of each nt present in each position of the splice site for the sequences present in that partition. The PFMs were transformed into position weight matrices (PWMs) by dividing each of the frequencies by the observed nt background frequencies and converting to a log scale [29]. A sliding window of nine nucleotides that contained a GT in the conserved donor splice site position was evaluated using the appropriate PWM (Figure 1). The score for each of these nine nt windows was equal to the sum of the corrected probabilities of each base in each position, as determined by the PWM (positions 1 and 2 were omitted because they were invariant, see Table 1). Only canonical donor sites were scored in both the *Purpuratus *and Vertebrate models.

**Table 2:** Characteristics of the sets of sequences from different taxa and groups of organisms

**Sequence Set^1^**	**# Sequences**	**# Positive Sites**	**# Negative Sites**	**Avg. # exons per gene**	**Avg. length (kb)**
Vertebrate	570	2079	149207	3.65	5.0
*Purpuratus *Model	292	2845	1142532	9.74	105.0
Vertebrate	2759	8599	1539898	3.12	10.3
*Purpuratus*	159	1714	684015	10.78	122.8
Protostome	1866	3303	361368	1.77	3.7
Experimentally					
Validated Echinoderm sequences	55	62	6228	1.13	2.2

^1^See Additional file 2, 3, and 4 for accession numbers of the sequences

### Determining model thresholds

**Figure 2:**
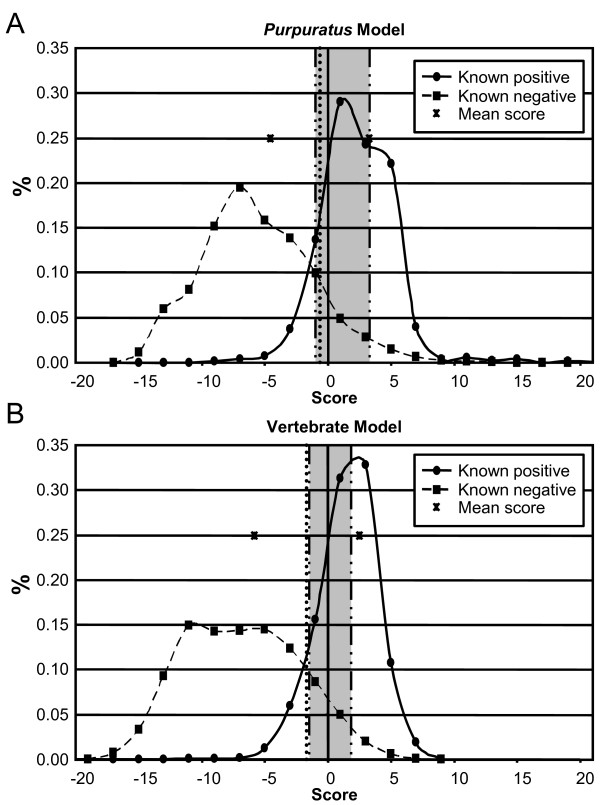
**Analysis of known positive and negative splice sites using the *Purpuratus *and Vertebrate splice site models**. Histograms of the scores given to known positive (solid lines) and negative (dashed lines) splice sites were generated (bin size = 2) for the *Purpuratus *(**A**) and Vertebrate (**B**) splice site models by analyzing the genes used to generate the models (Additional file 2, 3, and 4; [28]). For example, 22% of the known positive sites received scores between 4 and 6. The average of the means (Table 3) is shown by a vertical dotted line. The gray region corresponds to N_0.95_, and P_0.05 _(Table 3), which flank the left and right side of the gray region, respectively, and are shown as dashed/dotted lines. The ✳ located on the 0.25% line indicate the mean of the positive and negative scores.

The performance of each model was assessed using six measures (Equations 2–7; see Methods) that required binary classification of the sites (either positive or negative). Therefore, it was necessary to define a threshold for each model that separated the positive and negative scores. Histograms of scores from known positive and negative sites were created from the sequences that were used to generate the models (Figure 2). In a perfect model, there would be no overlap between the scores assigned to the positive and negative sites, because the positive sequences would all receive scores higher than the negative sites. This was not possible, however, due to the degeneracy of the splicing machinery and because certain sequences may act as donor splice sites in some sequences but are not functional in others. These factors complicate the results such that the scores for the positive and negative sites overlap. A threshold was required to define each score as either positive or negative for the model assessments. The ideal threshold would maximize the number of positives that were classified as positive (true positives; TP) and minimize the number of negative scores incorrectly labeled as positive (false positives; FP). A number of thresholds were analyzed, including the average of all the scores, the score at which 95% of the positive scores were considered positive (P_0.05_), and the score at which 95% of the negative scores were considered negative (N_0.95_; Table 3). The best threshold was the average of the means of the positive and negative scores (Figure 2) because it provided the most favorable balance between the number of true positives and false positives, and was therefore used in the subsequent evaluations of the models.

**Table 3:** Analysis of the sequences used to generate the models using the donor splice site models

	Model	
Characteristic	*Purpuratus*	*Vertebrate*

Min. Score	-15.029	-17.487
Max. Score	25.049	8.757
Avg. of Means^1^	-0.64	-1.64
		
P_0.05_^2^	-1.004	-1.491
N_0.95_^3^	3.329	1.843
Δ(N_0.95_, P_0.05_)^4^	4.333	3.335
# Possible Scores	49501	21561
% Possible Scores	15.77%	14.25%
		
Total Scores	313955	151286

^1^The average of the mean score for the known positive and known negative sites.

^2^The score at which 95% of known positive sites were positive (5% of positive sites were incorrectly called negative).

^3^The score at which 95% of known negative sites were negative.

^4^The difference between P_0.05 _and N_0.95_.

### Model performance

#### Donor splice sites in the S.  purpuratus sequences

**Figure 3:**
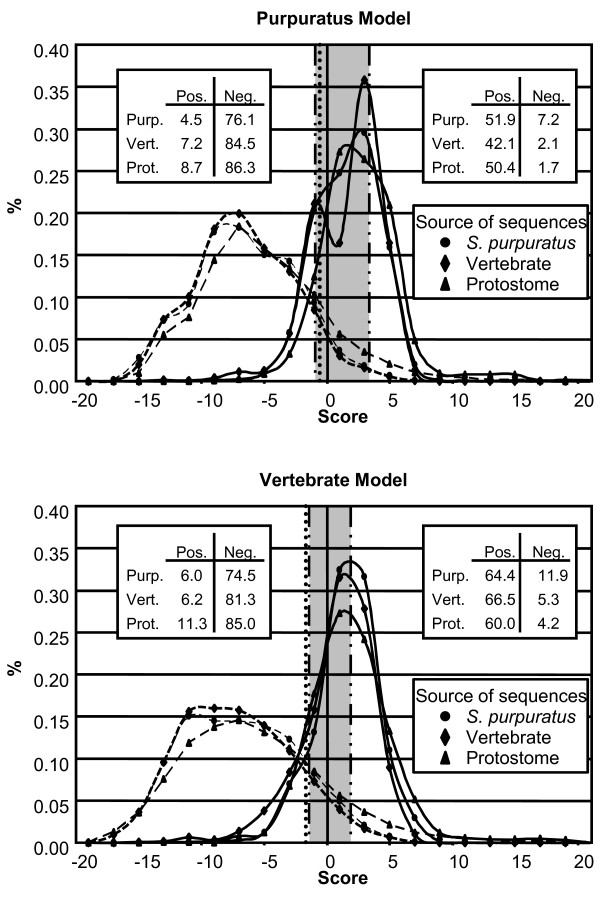
**Histograms to evaluate the models**. Genes isolated from *S. purpuratus *(circles), vertebrates (diamonds), and protostomes (triangles) were collected and analyzed using the *Purpuratus *(A) and Vertebrate (B) models. Histograms of the known positive (solid lines) and negative (dashed lines) donor splice sites were generated (bin size = 2). The average of the means (Table 3) is shown by a vertical dotted line. Values corresponding to N_0.95_, and P_0.05 _(Table 3) flank the left and right side of the gray region, respectively, and are shown as a dashed/dotted line. The tables within the graphs indicate the percentage of known positive (Pos.) and negative (Neg.) *S. purpuratus *(Purp.), vertebrate (Vert.), and protostome (Prot.) sequences, which were classified as positive or negative using the average of the means as the threshold.

The *Purpuratus *and Vertebrate donor splice models were used to evaluate an independent set of annotated *S. purpuratus *genes (Table 2). The frequencies with which the known positive and negative donor sites were predicted correctly were calculated using a 2 × 2 contingency table. The resulting numbers of TP, FP, true negative (TN), and false negative (FN) sites were used to calculate six measures of model accuracy. The *Purpuratus *model out-performed the Vertebrate model in five of the six assessments (Table 4; Figure 3). Most notably, the specificity (Sp) of the *Purpuratus *model was higher than that of the Vertebrate model, indicating that, compared to the Vertebrate model, more of the predicted positive sites were likely to be functional donor sites. The sensitivities (Sn) of the two models were approximately the same (Table 4). For each of the four assessments [correlation coefficient (CC), simple matching coefficient (SMC), average conditional probability (ACP), and approximate correlation (AC)] that combined Sp and Sn values, the *Purpuratus *model scored higher than the Vertebrate model (Table 4). Thus, when evaluating sea urchin sequences, the *Purpuratus *donor splice site model more accurately predicted whether or not a given sequence could be used as a splice signal.

**Table 4:** Evaluating the donor splice site models using three different sources of sequences.

Evaluation Sequences	Assessment*	*Purpuratus *Model	Vertebrate Model
*S. purpuratus*	Sp	0.054	0.045
	Sn	0.940	0.943
	CC	0.196	0.172
	SMC	0.786	0.738
	ACP	0.694	0.681
	AC	0.389	0.361
			
Vertebrates	Sp	0.037	0.026
	Sn	0.914	0.947
	CC	0.169	0.139
	SMC	0.868	0.804
	ACP	0.705	0.694
	AC	0.409	0.388
			
Protostomes	Sp	0.066	0.046
	Sn	0.894	0.892
	CC	0.225	0.181
	SMC	0.885	0.833
	ACP	0.711	0.692
	AC	0.422	0.385

* see Methods for definitions

#### Evaluating vertebrate sequences

Both models were also used to predict donor splice sites within vertebrate genes (Additional file 2). Unexpectedly, as when evaluating the sea urchin sequences, the *Purpuratus *model had higher Sp, CC, SMC, ACP, and AC than the Vertebrate model (Table 4; Figure 2). The Sn of the Vertebrate model was higher than that of the *Purpuratus *model, indicating that the Vertebrate model was able to identify more of the positive sites. When the vertebrate sequences were evaluated, the Sp and Sn values were lower than when the *S. purpuratus *sequences were evaluated by either model (Table 4).

#### Evaluating experimentally validated donor splice sites

A more extensive analysis of echinoderm splice sites has not been completed previously because, prior to the availability of the assembled *S. purpuratus *genome, insufficient numbers of gene sequences were available to analyze. As of January, 2008, only 55 echinoderm genes with annotated exons were deposited in GenBank. From these sequences, 62 canonical splice sites were experimentally validated (Table 2). These sequences were also used to test the two models for predicting donor splice sites. The *Purpuratus *model was able to identify 60 of 62 of the experimentally validated sites, while the Vertebrate model predicted 59 of 62 validated sites (Table 5). Furthermore, the Vertebrate model predicted more false positives than did the *Purpuratus *model. The *Purpuratus *model therefore had higher Sp and Sn, in addition to the assessment statistics CC, SMC, AC, and ACP. Thus, the *Purpuratus *donor splice site model is able to predict splice sites not only from annotated gene sequences, but also from experimentally validated sequences.

**Table 5:** Evaluation of the experimentally validated echinoderm sequences using the two donor splice site models.

		**Observed**			
		
		*Purpuratus Model*		*Vertebrate model*	
		Positive	Negative	Positive	Negative
**Prediction**	Positive	60	731	59	1037
	Negative	2	5497	3	5191
		
	Total	62	6228	62	6228
Assessments*	Sp	0.076		0.054	
	Sn	0.968		0.952	
	CC	0.253		0.204	
	SMC	0.883		0.835	
	ACP	0.731		0.710	
	AC	0.463		0.419	

*For a description of the assessments, see Methods. Higher values indicate greater accuracy. The range for all assessments is 0 to 1, with the exception of AC, which can range from -1 to 1.

#### Evaluating protostome sequences

As species that is phylogenetically positioned between vertebrates and insects, sea urchins contain homologues of genes from both protostome and deuterostome organisms [16]. It was therefore of interest to determine whether the *Purpuratus *donor splice site model would work better on other invertebrate sequences compared to the Vertebrate model. A set of protostome genes containing known splice sites was collected from NCBI and analyzed using the two models (Table 2). Similar to results comparing sea urchin and vertebrate genes, Sp and all four of the assessments that combined Sp and Sn were higher when the *Purpuratus *model was used to analyze the protostome genes compared to results using the Vertebrate model. The Sn values of the two models, however, were similar Figure 3; Table 4).

### Analysis of putative cryptic donor splice sites within the *185/333 *gene family

The 92 unique *185/333 *genes isolated from three animals [23] were used to analyze the donor splice sites with both the *Purpuratus *and Vertebrate splice donor site models. A total of 6743 GT sites were present within the sequences. The score for each GT site was categorized as "positive" (score ≥ N_0.95_), "negative" (score < P_0.05_), or "possible" (P_0.05 _≤ score < N_0.95_) (see Table 3). Because the aim of this study was to identify as many donor splice sites as possible, both the positive and possible scores were analyzed further as "candidate" sites. The position of each of these sites was mapped onto an alignment of the *185/333 *gene sequences [23,26] and the locations of the splice sites with respect to the positions of the elements were characterized. Because the elements have been defined based on the presence of gaps that optimized the alignments of messages [26] or genes [23], the element edges represented locations within the sequences in which at least one message contained a gap that could be the result of alternative splicing. Therefore, candidate donor splice signals located at the beginning of elements were of particular interest for further analysis.

#### Results from the *Purpuratus *model

Using the *Purpuratus *donor splice site model (Figure 1) to analyze the *185/333 *genes, 51 of the 6743 total GT sites were identified as positive and 1219 were classified as possible. On average, each of the 92 genes had 13.8 candidate donor splice sites. These sites were distributed among 51 positions within the alignment (Table 6). The known donor splice site (position 56) located at the 5' end of the intron received the highest average score for all of the genes analyzed (Table 6), and served as a positive control that supported the legitimacy of the model. Of the 1270 candidate donor sites, 18 were located within the intron and represented false positives because i) intron sequences have never been observed among any of the sequenced transcripts, and ii) translation of the known intron sequences lead quickly to a stop codon. Consequently, these 18 sites were not analyzed further. One candidate donor site (GT_7_) was present in all genes and was located within the first exon just after the starting methionine (position 7; Table 6). The first exon, which is 55 nt in length, encodes the hydrophobic leader [18,26]. If the GT_7 _site was a functional donor splice site, and worked with the known acceptor site at the 3' end of the intron, it would generate a transcript with an intact open reading frame without a leader sequence. Although this splice variant has not been observed among *185/333 *transcripts [18,25,26], alternative splicing using this donor site could theoretically affect the cellular localization of the encoded protein. Analysis of the 185/333 protein localization showed that most localized to vesicles in small and polygonal phagocytes and to the cell surface of small phagocytes, however, small amounts of 185/333 proteins were present in the cytoplasm of these coelomocytes [30].

**Table 6:** Location of candidate donor splice sites within the *185/333 *genes

		*Purpuratus *splice model			Vertebrate splice model		
Alignment Position^1^	Element^1^	Number of Sequences with positive sites^+^	Average Score	Number of Unique sites	Number of Sequences with positive sites^+^	Average Score	Number of Unique sites
7	Leader	92	0.9141	2	92	0.3802	2
56	Leader	92	3.6544	2	92	2.0761	2
662	Ex2	38	-0.8232	1	84	0.522	2
697	Ex2	2	3.6456	1	.	.	.
698	Ex2	.	.	.	24	0.9431	1
731	Ex3	3	2.116	1	.	.	.
738	Ex4	38	-0.9527	1	.	.	.
758	Ex4	.	.	.	74	0.3271	1
791	Ex4	1	0.3319	1	1	-0.2099	1
806	Ex4	51	2.1556	1	.	.	.
813	Ex5	3	-0.7904	1	.	.	.
888	Ex5	2	-0.7852	1	.	.	.
956	Ex5	5	2.1018	1	.	.	.
1001	Ex7	.	.	.	73	-0.2442	1
1031	Ex7	.	.	.	1	0.7604	1
1034	Ex7	3	-0.0971	1	3	-0.5625	1
1043	Ex7	.	.	.	90	-0.8223	1
1082	Ex7	92	-0.574	2	.	.	.
1103	Ex9	80	-0.0487	2	53	-0.8249	1
1107	Ex9	2	1.0598	1	2	-1.1971	1
1127	Ex10	.	.	.	2	-1.4636	1
1136	Ex10	11	0.4482	1	11	-0.5977	1
1148	Ex11	2	-0.6091	1	2	3.2707	1
1235	Ex13	34	-0.958	1	79	1.3481	3
1271	Ex13	76	-0.8621	2	.	.	.
1397	Ex16	38	-0.9555	1	38	1.4972	1
1406*	Ex17	35	-0.9532	1	35	1.1479	1
1412	Ex17	1	1.5242	1	1	-1.1572	1
1448	Ex17	3	-0.2399	1	3	1.3501	1
1464	Ex17	.	.	.	34	1.8815	1
1478*	Ex18	38	-0.3842	1	.	.	.
1529	Ex19	84	-0.8774	2	84	1.5862	2
1538	Ex19	29	-0.8506	1	29	1.2722	1
1646*	Ex22	79	-0.3887	3	.	.	.
1697	Ex23	.	.	.	2	1.4733	1
1895	Ex23	2	-0.1887	1	.	.	.
1946	Ex23	.	.	.	2	1.4733	1
2063	Ex24	14	-0.3657	1	.	.	.
2153	Ex25	92	3.1818	1	92	2.0725	1
2171	Ex25	92	2.4747	3	92	0.5568	3
2183	Ex25	.	.	.	92	1.4031	1
2215	Ex25	17	-0.8872	1	.	.	.
2287	Ex25	2	-0.0698	2	89	1.0827	6

^1^See [23]

*Indicates that the GT is located at the beginning of the element

^+^Total number of genes analyzed was 92.

The remaining 31 candidate donor splice sites were located within the second exon, the majority of which were within elements rather than at their edges. The positions of splice sites were analyzed with respect to elements from the two previously published alignments [23,26]. Although no splice sites were found at the edges of elements from the repeat-based alignment, three sites, were located at the 5' end of three different elements from the cDNA-based alignment (Table 6). The GT in positions 1406, 1478, and 1646 were the first two nt of elements Ex17, Ex18, and Ex22, respectively. This is noteworthy because elements were defined based on gaps present within the transcript sequences [26]. If these sites were used as donor splice sites, this would introduce a gap in the transcripts starting with the GT site. GT_1406 _was located at the 5' end of Ex17, an element that was present in all transcripts except those missing subelement Ex15a [25,26]. However, those genes that had Ex15a also lacked Ex17 [23], which suggested that the absence of Ex17 among these transcripts was due to its absence within the corresponding genes, rather than to mRNA splicing. GT_1478 _was located at the 5' end of Ex18, which is only 30 bp long, and when it was absent from transcripts, Ex19 was always present [25,26]. Species-specific minimal intron sizes in humans, *Arabidopsis thaliana, Drosophila melanogaster*, and *Caenorhabditis elegans *are 92, 89, 61, and 48 bp, respectively [31], and Ex18 is much shorter. Thus, although the minimal intron size in *S. purpuratus *is unknown, the size of Ex18 suggested that it would not be spliced out of a *185/333 *transcript without splicing out neighboring elements. However, the last two nt of Ex18 are CG, which may function as an alternative acceptor site. Although GT-CG splicing has been observed, it is believed to account for less than 0.2% of functional splice sites [32]. GT_1646 _was located at the beginning of Ex22, however, this element is present in all transcripts that also contain the preceding element Ex21. This suggested that, although GT_1646 _could theoretically be used by the splicing machinery, splicing at this site has not been observed among the 687 *185/333 *transcripts that have been characterized to date [18,25,26]. Therefore, although possible cryptic donor splice sites were identified within the *185/333 *gene sequences using the *Purpuratus *splice site model (Figure 1), it was unlikely that any of these sites were used for intragenic splicing to generate element pattern variation based on comparison of the known *185/333 *transcript and gene sequences. This is in agreement with previous data that suggested that the majority of the transcript element pattern variation was encoded by the second exon in this diverse gene family [23].

#### Analysis of the *185/333 *genes with the Vertebrate model

The *185/333 *gene sequences were also analyzed using the Vertebrate model that was generated based on vertebrate sequences. Among the 6743 sites, 244 were characterized as positive using the Vertebrate model, and 1186 were classified as possible, for a total of 1430 total candidate donor splice sites that were located in 50 positions within the alignment (Table 6). The known donor splice site in position 56 located at the 5' end of the intron was identified in all 92 genes, although this site did not have the highest average score (Table 6). In agreement with the *Purpuratus *model, 19 candidate sites were identified within the intron, and GT_7_was also identified as a candidate donor splice site in all of the genes. The 30 remaining sites were not located at the 5' edge of elements, and were therefore considered unlikely to be utilized as donor splice sites.

Together, the two splice site models identified 67 candidate donor splice sites among the *185/333 *genes. It was surprising that only 34 of the sites were recognized by both models. This may suggest that the remaining 33 sites were false positives, although there was no significant difference between the scores of the sites that were recognized by both models vs. those that were recognized by just one. The fact that the two models scored sites differently supports the idea that the two models recognize different sequences, which may hint at evolutionary differences between optimal splice sites in different groups of animals.

### Do linked *185/333 *genes undergo intergenic splicing?

The *185/333 *genes are closely linked, some as close as ~3 kb [23]. Therefore, it is possible that a primary transcript could be generated that spans two *185/333 *genes followed by splicing the intergenic region using cryptic or alternative donor splice sites to produce a hybrid message that resembles the typical *185/333 *messages transcribed from single genes. To identify transcripts that might result from this type of putative intergenic splicing, the genes and messages from individual animals were analyzed [24]. If a message sequence was most similar to one gene 5' of a candidate splice site and most similar to a different gene 3' of that splice site, this would provide evidence for intergenic splicing. Because the GT site would be removed during the splicing process, the only candidate splice sites within the alignment that were analyzed, were those in which gene sequences had a GT and message sequences had a gap. Only two sites, GT_1406 _and GT_1478 _(Table 6), met the criteria for further analysis.

GT_1406 _was located at the beginning of element Ex17 (Additional file 1B). There was only one message in which Ex16 was present and Ex17 was absent (4–2406; GenBank accession number EF065721), although 35 of the genes contained a high scoring candidate donor splice site at this position. To identify putative intergenic splicing, the sequence prior to and following GT_1406 _from message 4–2406 was compared to all of the known *185/333 *gene sequences isolated from the same animal and scored for substitutions and indels as in [24]. Previous analysis of this message suggested that the gene from which it was mostly likely transcribed was unknown because the message was not significantly similar to any of the genes [24]. If the 5' and 3' ends of the message were similar to two separate genes, a more likely scenario may be that message 4–2406 is the product of intergenic splicing such that the region 5' of GT_1406 _was transcribed from one gene, and the 3' end of the message from a different gene. A more detailed sequence analysis showed that the region 5' of GT_1406 _was most similar to gene 4–1544 (GenBank accession number EF607785), but differed by seven indels and 49 substitutions. On the other hand, analysis of the 3' end of message 4–2406 showed that it differed from gene 2-036 (GenBank accession number EF607718) by only a single nucleotide (Additional file 1B). One interpretation of this result may be that message 4–2406 was generated from transgene splicing from an unknown gene to gene 2-036, although other interpretations, such as gene recombination, are possible [22].

The other candidate donor site of interest, GT_1478_, was located at the beginning of element Ex18. A total of 38 genes contained GT_1478 _(Table 6), and there were four messages in which the position was located at the beginning of a gap (4–2433, 4–2441, LPS2-2405, and LPS2-2412). The sequences from these messages located 5' and 3' of GT_1478 _were compared to the corresponding regions of the genes, and the gene from which each end of the messages was most likely transcribed was determined [24]. This was done by adding the lowest score from the 5' and 3' ends and comparing that to the score of a full-length gene sequence [24]. For each of the four messages, the score of the full-length gene from which the message was likely to have been transcribed was lower than the sum of the scores from the two halves. This indicated that it was more probable that the message was transcribed from a single gene, rather than being the product of intergenic splicing. Therefore, although it was theoretically feasible that some of the *185/333 *messages may be result of splicing between two neighboring genes, analysis of the candidate splice sites within the gene and message sequences and comparison of the sequence on either side of these splice sites support the theory that each message was most often the product of a single gene.

## Discussion

The data presented here demonstrate that a splice site model generated using sea urchin sequences can predict more accurately the donor splice sites in sea urchin and protostome sequences than a model generated using vertebrate sequences. It was intriguing that the *Purpuratus *donor splice site model also worked better on the vertebrate sequences than did the Vertebrate model. The accuracy of the *Purpuratus *model was lower when analyzing sea urchin sequences than when analyzing the vertebrate sequences. The *Purpuratus *model was better able to predict the splice sites in the set of experimentally validated echinoderm sequences. Although putative cryptic donor splice sites were identified within the *185/333 *gene sequences, searches of the currently available transcript sequences did not show that these splice sites were generally employed to remove elements and change the element pattern, although one example was identified that may employ trans-gene splicing to generate novel messages.

### Analysis of the splice site models

One problem with the *Purpuratus *donor splice site model is that it has low overall specificity, which predicts many false positives. The reason for this is that the nine nt sequence that includes the donor splice site could be a functional donor splice site in some cases and a non-functional donor splice site in others. For example, of the total 313,955 GT sites that are present in the set of *S. purpuratus *sequences used to generate the *Purpuratus *model, there are only 16,247 unique nine nt sequences (this number of sites is possible given all combinations of the four nucleotides in each of the seven variable positions of the splice site). Of those, 723 nine nt sequences are both functional and non-functional in different sequences. This is the basis for the overlap of the histograms of the positive and negative scores, and confounds the establishment of a clear threshold to define positive scores. A number of thresholds were examined in this analysis, and it is feasible to continue to adjust the threshold of positive scores based on the aims of this and future studies. For example, in this analysis of *185/333 *sequences, the aim was to identify as many putative splice sites as possible. Consequently, the threshold was defined as P_0.05 _because this was the level above which 95% of known positive scores fell. However, this threshold could be raised if a more stringent analysis is required. Furthermore, because this analysis considers only splice sites and does not evaluate whether or not a sequence is likely to be a coding region, this method is not likely to be optimal for *ab initio *gene prediction, but rather, is better suited to identify cryptic or alternative donor splice sites within the context of known genes in conjunction with other data.

### Acceptor site analysis

Acceptor sites are much more complex and the sequences of acceptor sites are much more degenerate, and therefore more difficult to model [14,28]. During splicing, the deleted intron forms a lariat structure, which is part of the process to ligate the adjoining exons. Lariat formation is mediated by the branch point region, a short sequence that is located between 10 and 50 nt upstream of the acceptor site [33]. Previous studies have shown that the sequence conservation of this branch point is very low. For example, the consensus branch point sequence (YYRAY) has been found in the correct position with respect to the acceptor site in only 30% of vertebrate sequences analyzed [28]. Despite the lack of conservation in either the branch point sequence or its location, a strong pyrimidine bias has been observed in the region upstream of the acceptor site, as introns commonly end with a poly-pyrimidine stretch [14,34]. Although this bias was exploited to generate a simple model that predicts acceptor splice sites, no predicted acceptor sites were identified within the *185/333 *sequences other than the known site (data not shown). Given the difficulty of modeling acceptor sites, and the lack of trans-gene or alternative splicing within the *185/333 *sequences, this was not explored further.

### The evolution of splice sites

Although little attention has been paid to splicing machinery in sea urchins, the small nuclear RNA ribonucleoprotein (snRNP) particles have been characterized [35]. Gene models corresponding to components of the spliceosome, including the U5 snRNP protein (SPU_003407), U11/U12 snRNP protein (SPU_026605), and the U4/U6.U5 tri-snRNP-associated protein (SPU_019522) have been annotated in the sea urchin genome [16]. Furthermore, analysis of transcripts expressed in sea urchin coelomocytes in response to LPS shows a number of mRNAs that encode proteins involved in RNA splicing [18]. These include the ET putative translation product, nuclear RNA- and DNA-binding proteins, heterogeneous nuclear ribonucleoprotein R, splicing factor 30, and a conserved nonhistone nucleic acid-binding protein. This suggests that coelomocytes responding to an immune challenge have increased gene expression that requires a coordinated increase in the expression of proteins involved in RNA splicing and processing. The slight differences observed in the frequency of nt in the putative U1 binding site between the *S. purpuratus *donor sites and the vertebrate donor splice sites, as well as the differences in the non-adjacent dependencies between the sequences from the different phyla imply differences in the recognition specificity of the spliceosome proteins. The most notable difference between the two models is that the consensus sequences differ at the +3 position, where Purpuratus has a consensus A, whereas the vertebrate model has an A/G [28]. This difference suggests that the Purpuratus model is more stringent, and therefore more specific, which agrees with the hypothesis that alternative splicing evolved by gradually relaxing the splice sites (see [36]). Phylogenetic analyses of the proteins involved in RNA splicing and their active sites may shed additional light on these differences.

### Analysis of the *185/333 *splice sites

Although candidate donor splice sites are present within the *185/333 *gene sequences, no transcripts have been identified that use these splice sites. Although some of the donor splice sites are located at the 5' edge of elements, these elements are present in similar frequencies in both genes and messages, suggesting that they are not spliced out and do not alter element pattern diversity. Furthermore, no hybrid transcripts were identified that appeared to be composed of two different genes, which decreased the possibility that trans-gene splicing is involved in diversification of the *185/333 *transcripts. The *185/333 *gene family has not been exhaustively sampled [23], consequently it is possible that a pair of genes from which a corresponding hybrid transcript might be spliced may yet be identified. However, our data strongly suggest that the *185/333 *system is not using cryptic splice sites for diversification of the messages.

## Conclusion

The data presented here are the first published analyses of echinoderm splice sites, and the first splice site models generated from echinoderm sequences. As more invertebrate genomes are sequenced, the utility of these types of models will increase for identifying cryptic or alternative splice sites within newly annotated genes. Because most of the available data stems from mammals, the splice site models that have been available to date have been tailored towards those sequences. Only the *Purpuratus *donor splice site model was able to identify putative splice sites located at the edges of the known *185/333 *elements, which suggests that the Vertebrate splice site models are insufficient for sea urchin sequence analysis. It was of note that the *Purpuratus *donor splice site model also predicted protostome splice sites with greater accuracy than the Vertebrate model. The procedure for generating this model is straightforward, and could be repeated using sequences from any species or phylum, given enough available sequences.

Previous work has suggested that there are two levels of diversification of the *185/333 *system. First, the *185/333 *genes may undergo an elevated rate of gene recombination, which may be promoted by the numerous repeats that are present both within and between the genes [22]. Second, the low correspondence between the sequences of the genes and the messages from individual sea urchins suggests that the messages are edited, perhaps by a constitutive cytidine deaminase [24]. The models described and tested here have been used to search for a possible third level of diversification of the *185/333 *messages through alternative and trans-gene splicing using cryptic splice sites. The results suggest that, unlike the *Dscam *gene in arthropods [37-39], the sea urchin *185/333 *system either does not employ alternative or trans-gene splicing, or that the occurrences are below our abilities to detect them. This highlights how different groups of organisms use different approaches to accomplish immune diversification.

## Methods

### Sequence data

A summary of the characteristics of the sequences used to generate and evaluate the models is found in Table 2. The sequences used to establish the positive threshold for the Vertebrate model were taken from [28]. Vertebrate and protostome sequences for evaluation were downloaded from NCBI . Accession numbers and coordinates are shown in Additional files 2 and 3, respectively. The set of experimentally validated echinoderm gene sequences was downloaded from NCBI and used to assess the splicing models. Accession numbers and coordinates of these sequences are given in Additional file 4.

### Generating the donor site models

For each position within the donor site, the frequency of each nucleotide was calculated (Figure 1A). The most common nucleotide was considered the consensus. When the consensus nucleotide was present in less than 50% of the sequences, the two most frequent nucleotides were both considered consensus (see position -3; Figure 1A). To identify non-adjacent dependencies between the consensus indicator variable, *C*_*i*_, and the nucleotides in position *X*_*j*_, the χ^2 ^value was calculated for *C*_*i *_vs. *X*_*j*_, for all pairs of *i, j *such that *i *≠ *j*. The dependencies were calculated such that if the nucleotide indicator (*X*_*j*_), matched the consensus nucleotide (*C*_*i*_), the score was 1; otherwise, they received a score of 0. Positions 1 and 2 (the GT site) were omitted, as they were invariant in this data set. Using the χ^2 ^values determined above, for each position *i*, the sum

(1)

was calculated. The position *i*, with the greatest value of *S*_*i *_was used to subdivide the set of splice sites into those containing the consensus nucleotide at position *i*, and those containing a non-consensus nucleotide. The process was repeated until one of the three following conditions occurred: (1) no more subdivisions were possible because all of the positions had been used; (2) no significant dependencies were observed; or (3) the subdivided datasets became too small as to no longer provide reliable data (< 500 sequences). The models were implemented using PERL scripts (Additional files 5 and 6).

### Measures of assessing the donor site models

The accuracy of the models was assessed using six measures: sensitivity (Sn), specificity (Sp), correlation coefficient (CC), simple matching coefficient (SMC), average conditional probability (ACP), and approximate correlation (AC). The formulas and rationales for each of these parameters are described in [5,28]. Briefly, for each of the models, a 2 × 2 contingency table was generated to identify the number of true positives (known positive sites that were scored as positive; TP), false positives (known positive sites that were scored as negative; FP), false negatives (known negative sites that were scored as positive; FN), and true negatives (known negative sites that were scored as negative; TN) (Figure 3). Sn and Sp are the most general measures of model accuracy, defined such that

(2)

measured how many of the true positives the model was able to identify, and

(3)

measured how many of the positives predicted by the model were true positives. Although these measures provided some insight into the accuracy of the models, it was of interest to use a single value that incorporated both Sn and Sp to better compare the performances of the models. The CC is a measure traditionally used by gene prediction programs [5], and is defined as

(4)

The CC measures the probability of a GT donor site being predicted as positive given that it actually is positive and being predicted as negative given that it actually is negative. As noted by [5], the major flaw in the utility of CC is that it is undefined when any of the values on the denominator is equal to 0. That is, when, either reality or prediction lacks both positive and negative donor splice sites. To circumvent this problem, the SMC is used, where

(5)

SMC is a measure of the probability that a given GT site is correct, i.e., that it is assigned the same value (positive or negative) in both reality and prediction. ACP is another single scalar value that assesses the global accuracy of the models using the 2 × 2 contingency table, as does CC, but can be calculated in any circumstance, like SMC. ACP is defined as

(6)

[40], and can be interpreted as the probability that a GT site is in a given state. ACP is a probability that ranges from 0 to 1, but is transformed into the AC through

(7)

such that the AC ranges from -1 to 1 and can be compared to the CC and can be computed in any circumstance.

## Authors' contributions

KMB was responsible for and prepared the manuscript. LDF provided invaluable guidance in the generation and analysis of the models. LCS directed the research, edited the manuscript and provided funding. All authors read and approved the final manuscript.

## Supplementary Material

Additional file 1 **Representative *185/333 *element patterns**. An illustration, modified from [23] that illustrates the element patterns of the *185/333 *messages and the locations of the putative donor splice sites. Click here for file

Additional file 2 **Vertebrate sequence accession numbers**. An Excel spreadsheet containing the accession numbers of the vertebrate sequences used to evaluate the splicing models. Click here for file

Additional file 3 **Protostome sequence accession numbers**. An Excel spreadsheet containing the accession numbers of the protostome sequences used to evaluate the splicing models. Click here for file

Additional file 4 **Echinoderm sequence accession numbers**. An Excel spreadsheet containing the accession numbers of the experimentally validated echinoderm sequences used to evaluate the splicing models. Click here for file

Additional file 5 ***Purpuratus *splicer**. A PERL script to implement the *Purpuratus *splicing model. Click here for file

Additional file 6 **Vertebrate splicer**. A PERL script to implement the Vertebrate splicing model. Click here for file
