# Supplementary material for: A method for identifying alternative or cryptic donor splice sites within gene and mRNA sequences. Comparisons among sequences from vertebrates, echinoderms and other groups
Source: BMC Genomics. 2009 Jul 16;10:318. doi: 10.1186/1471-2164-10-318 (PMC2721852; doi:10.1186/1471-2164-10-318)

**Additional File 1: *185/333* element patterns.** A. The *elements* of the *185/333* genes and messages are defined by the gaps inserted to optimize the alignment (modified from [23]). The *element patterns* are defined by the mosaic presence or absence of elements (shown as colored boxes). The locations of the two exons are shown, while the intron is omitted. The black arrows indicate the positions of the possible donor splice sites shown in Table 6, and the red arrows indicate positions at the beginning of elements. For a full description of the *185/333* gene and message elements and element patterns, see [23-26]. B. Message 4-2406 may be the product of transgene splicing between gene 2-036 and another gene. The GT_1406_ putative donor splice site is indicated with a red arrow. SNPs between the two sequences are indicated by black arrows. The region 5’ of the GT_1406_ site is not significantly similar to any known *185/333* gene sequence. However, the region 3’ of GT_1406_ is identical to gene 2-036, with the exception of one SNP in element 25b.


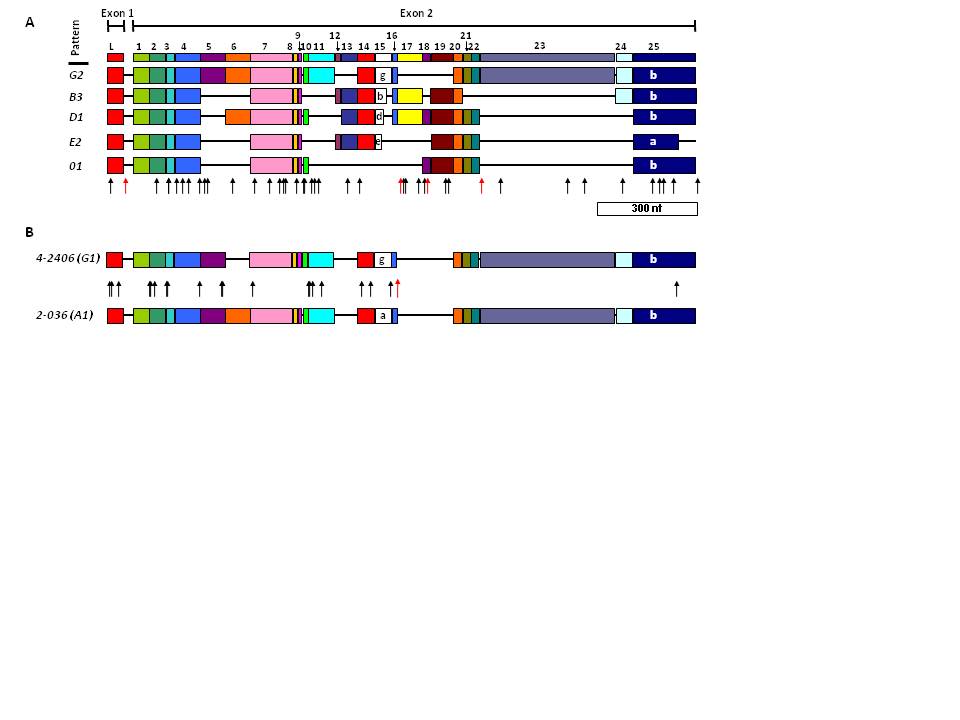

Supplement: Additional file 1 — Representative 185/333 element patterns. An illustration, modified from [23] that illustrates the element patterns of the 185/333 messages and the locations of the putative donor splice sites. [file 1471-2164-10-318-S1.docx]
